# Exploration of sensory-motor tradeoff behavior in Parkinson’s disease

**DOI:** 10.3389/fnhum.2022.951313

**Published:** 2022-10-25

**Authors:** Sonal Sengupta, W. Pieter Medendorp, Luc P. J. Selen, Peter Praamstra

**Affiliations:** ^1^Donders Institute for Brain, Cognition and Behaviour, Radboud University Nijmegen, Nijmegen, Netherlands; ^2^Department of Neurology, Radboud University Nijmegen Medical Centre, Nijmegen, Netherlands

**Keywords:** Parkinson’s disease, sensorimotor function, sensorimotor tradeoff, motor inhibition, optimal control

## Abstract

While slowness of movement is an obligatory characteristic of Parkinson’s disease (PD), there are conditions in which patients move uncharacteristically fast, attributed to deficient motor inhibition. Here we investigate deficient inhibition in an optimal sensory-motor integration framework, using a game in which subjects used a paddle to catch a virtual ball. Display of the ball was extinguished as soon as the catching movement started, segregating the task into a sensing and acting phase. We analyzed the behavior of 9 PD patients (ON medication) and 10 age-matched controls (HC). The switching times (between sensing and acting phase) were compared to the predicted optimal switching time, based on the individual estimates of sensory and motor uncertainties. The comparison showed that deviation from predicted optimal switching times were similar between groups. However, PD patients showed a weaker correlation between variability in switching time and sensory-motor uncertainty, indicating a reduced propensity to generate exploratory behavior for optimizing goal-directed movements. Analysis of the movement kinematics revealed that PD patients, compared to controls, used a lower peak velocity of the paddle and intercepted the ball with greater velocity. Adjusting the trial duration to the time for the paddle to stop moving, we found that PD patients spent a smaller proportion of the trial duration for observing the ball. Altogether, the results do not show the premature movement initiation and truncated sensory processing that we predicted to ensue from deficient inhibition in PD.

## Introduction

Suppressing unwanted movements is essential for goal-directed action. There is a growing body of work which suggests dysfunctional inhibition of unwanted movements in Parkinson’s disease (PD) patients (Gauggel et al., 2004; Seiss and Praamstra, 2004; Wylie et al., 2005; Jahanshahi et al., 2015a). Inhibition in PD has been studied mostly using conflict or stop tasks. In typical conflict tasks, stimuli have two conflicting dimensions: One dimension that the subject is instructed to respond to and one that is irrelevant (e.g., required movement direction and spatial location of stimulus in the anti-saccade and spatial stimulus-response compatibility task). The irrelevant dimension interferes with the processing of the task-relevant dimension by engaging pre-potent response tendencies (Stroop, 1935; Simon and Small, 1969; Eriksen and Eriksen, 1974; Munoz and Everling, 2004). Stop tasks by contrast, such as the stop signal task and the go-nogo task, use explicit signals that instruct subjects to abort (preparation of) a movement signaled by a preceding stimulus or by the expectation of a go stimulus (Logan and Cowan, 1984). A role of the basal ganglia in these different forms of behavioral inhibition is well-established, in agreement with PD patients’ abnormal performance on these tasks (Jahanshahi et al., 2015b).

Parkinson’s disease patients’ reduced inhibition in conflict and stop tasks is commonly regarded as a key element of impaired executive function or cognitive control (Dirnberger and Jahanshahi, 2013; Robbins and Cools, 2014). As phrased by Jahanshahi et al. (2015b): “….inhibition, disinhibition and facilitation are the essence of executive control — that is, the ability to modify behavior, depending on context or environmental demands, to achieve specific goals.” However, as suggested by Aron (2007), the notion of inhibition is probably overextended in the area of cognitive control; the operation of an actual inhibitory mechanism is confined to the motor domain. The inhibitory mechanism Aron (2007) refers to concerns a mechanism for behavioral inhibition, relying on fronto-subthalamic circuitry, called upon in stop tasks, as evidenced in neuro-imaging and neurophysiological studies (Eagle and Robbins, 2003; Aron, 2006; Coxon et al., 2006; van den Wildenberg et al., 2006). Mindful of the proposition that true inhibition, in a mechanistic sense, is motor inhibition, we sought to address the question whether PD patient’s deficient inhibition in rather artificial conflict and stop tasks, also compromises their performance in more naturalistic sensory-motor tasks. In the present paper we approach this question by means of a virtual ball-catching experiment (Faisal and Wolpert, 2009; Sengupta et al., 2018). Note that natural motor behavior, such as gait and prehension, is of course widely investigated in PD. Also, associations between deficient inhibition and (freezing of) gait are studied. However, relevant work in this area is rarely directed at the question how impaired inhibition affects natural motor performance, as our study aims (but see Georgiades et al., 2016).

There are two important reasons why a ball catching task might be well suited to study motor inhibition in PD patients. First, previous studies have shown that patients initiate their movements earlier and move significantly faster to catch a moving ball compared to when reaching to a static ball (Majsak et al., 1998, 2008; Bieńkiewicz et al., 2013, 2014). Second, the ball catching task has an embedded implicit time constraint, adding urgency for action and thus facilitating movement in PD patients (Ballanger et al., 2006, 2008; Majsak et al., 2008; Fooken et al., 2022). However, the facilitation by the perception of movement and by urgency, might in fact be a manifestation of reduced inhibition. Based on this hypothesis, we expected poorer performance under the conditions in our task, in which we manipulated visual feedback. Specifically, we extinguished the display of the ball as soon as subjects started to move, dividing the task in a sensing and an acting stage (Faisal and Wolpert, 2009; Sengupta et al., 2018). A tendency of patients to respond prematurely, due to deficient inhibition, would therefore curtail the sensory sampling and thus compromise performance.

Analysis of subjects’ performance was not limited to describing catching performance and reaction time (i.e., switching time between sensing and acting), but also involved a modeling analysis from the perspective of an optimal control framework (e.g., Körding and Wolpert, 2006). The brain has been shown to use statistical knowledge of sensory and motor uncertainty to optimize performance (Trommershäuser et al., 2005; Battaglia and Schrater, 2007). In our experiment, subjects’ sensory and motor uncertainty were assessed independent of the main task and used to evaluate whether, in the main task, switching times were chosen in such a way as to produce the optimal tradeoff between sensing and acting. Note that, in theory, PD patients might produce short switching times not as an expression of disinhibition but to allow more time for movement, given their presumably greater motor uncertainty. Thus, the optimal control framework provided analysis tools that helped assess whether early, possibly disinhibited movement initiation was detrimental to goal directed action or served a compensatory role.

Our main analyses indicate that PD patients do not initiate movements earlier than controls. Both patients and controls could withhold their movements to optimize their behavior. However, exploration of the movement kinematics showed smaller peak velocity for the patients, but higher velocity at the time of interception. In effect, patients elongated the predefined 1.4 s trial duration by having non-zero velocity at the time of interception, whereas controls shortened the trial and were waiting for the ball to land. Redefining the trial duration to the total movement time, patients spent a smaller proportion of the trial for observing the ball. We also analyzed the temporal variability of movement initiation, which was compromised in patients. That is, while controls show clear modulation of variability in timing of movement initiation, based on the task constraints, patients did not. Based on this result, we suggest that the behavior of the patients reflects a deficiency in generating exploratory behavior for optimizing goal-directed movements.

## Materials and methods

### Subjects

Ten naïve right-handed PD patients [eight male, aged 63 ± 5 years (SD)] with normal or corrected-to-normal vision performed the experiment. Patients were mildly to moderately affected and on their regular medication during the experiments. The control group consisted of 10 healthy subjects (nine male, aged 64 ± 4 years), who reported no history of psychiatric, neurological or musculoskeletal disorders. All subjects received detailed verbal and written information about the experiments and provided written informed consent before participating. Experiments were approved by the local ethics committee of the Faculty of Social Sciences, Radboud University Nijmegen, The Netherlands. All subjects were offered compensation for their time (€ 10/h).

### Set-up

**FIGURE 1:**
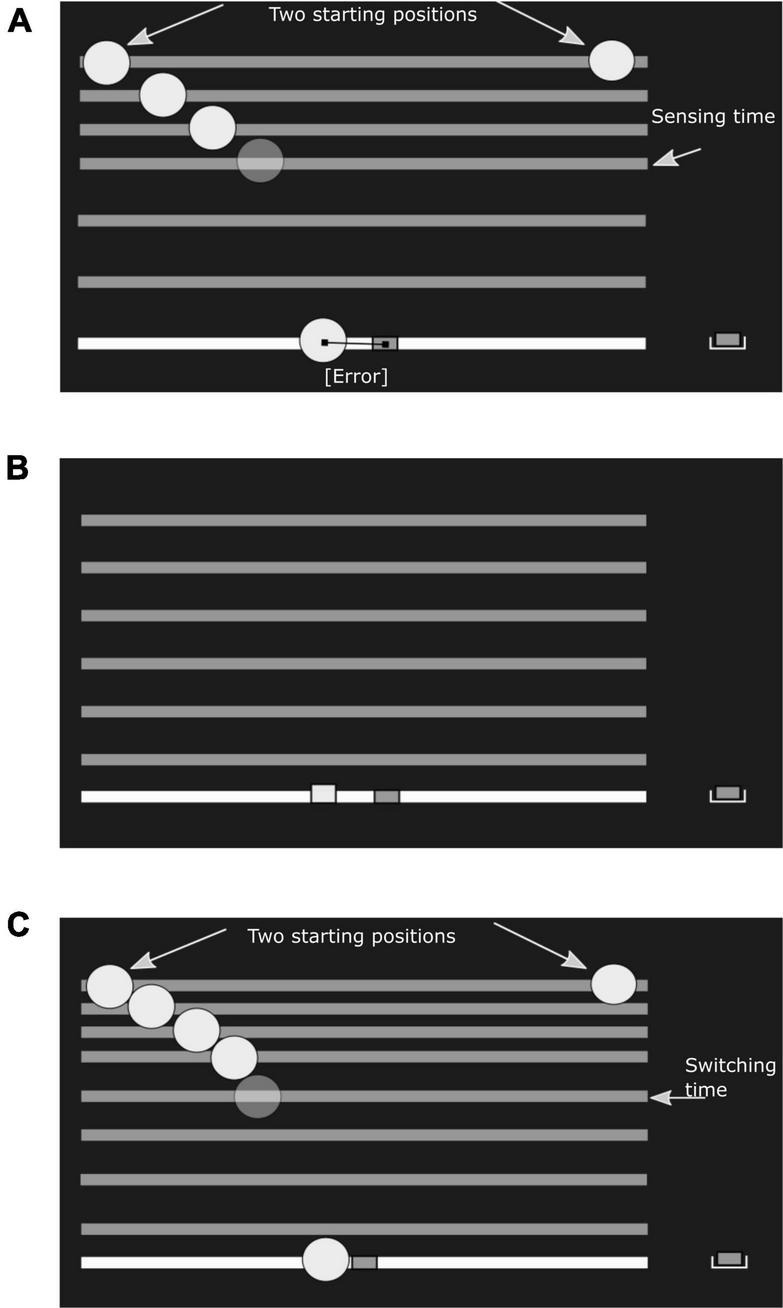
Experimental conditions. In the experiment the ball was drawn in green and the paddle in red. Ground level, the falling bar and the paddle start location were white. **(A)** Sensory task: The subject would see a ball falling, with a constant horizontal velocity and under simulated gravity, from either the left or right start position (fixed within a session). The horizontal bar would fall along with the ball. After a pre-determined sensing time, the ball disappeared, but the bar kept falling. After the bar hit ground level, the subject had infinite time to indicate the estimated landing position, using the paddle. **(B)** Motor task: A reach target is shown as a small red square at ground level. The required movement time is first indicated by a bar falling at constant velocity. Next, the subject has to reach from the start position to the target within the prescribed movement time, again with the bar falling as an indicator of remaining time. **(C)** Combined task: The ball falls from either the left or right start position (fixed within a session). The ball disappears once the subject starts moving the paddle, defining the ‘switching time’. The bar continues to fall to function as a timer. The task of the subject is to catch the ball on the paddle before it hits ground level. In all conditions the subject receives feedback about the final paddle position relative to the true landing position of the ball **(A,C)** or reach target **(B)**.

Subjects were seated in front of a planar robotic manipulandum (vBOT, Howard et al., 2009). They used their right hand to make reaching movements while leaning slightly forward and looking into a semi-silvered mirror reflecting the visual scene projected from a monitor suspended above (Asus, model VG278H), with their forehead resting against a headrest. The arm was not visible, but the position of the hand was veridically indicated in the working plane as a rectangle which functioned as a paddle during the experiment (see Figure 1). Movement of the paddle was restricted onto a line parallel and 30 cm in front of the shoulders by simulating a virtual channel (Scheidt et al., 2000) with the manipulandum. For a more extensive description of the setup see Sengupta et al. (2018).

### Paradigm

**FIGURE 2:**
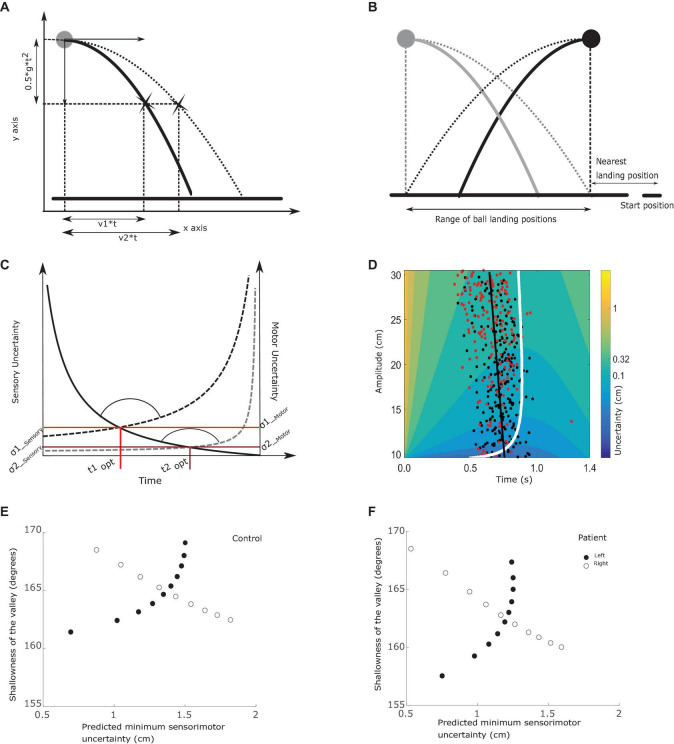
Relations between outcome measures. **(A)** Trajectory uncertainty. The ball is projected with a horizontal velocity v1 (between 0 and 15 cm/s) and no initial vertical velocity. The vertical acceleration is set to 25 cm/s^2^. After *t* seconds, the ball will have traveled v1*t cm horizontally and 0.5*g*t2 vertically. Since the starting height is fixed, the ball always reaches ground level after 1.4 s. Subjects can only move the paddle horizontally at ground level and need to estimate where the ball will hit the ground based on sensory information about the ball trajectory. Sensory uncertainty, at-least in this design, depends on the initial horizontal velocity of the ball; with increasing velocity sensory uncertainty increases. **(B)** Starting positions. The ball could start from two positions, 20 cm apart, whereas the hand always started moving from the bottom right corner. The horizontal black line represents the ground level. The closest landing position was 9 cm from the hand starting position and was associated with 0 cm/s or 15 cm/s horizontal velocity. In the figure, we obscured a portion of the trajectories to give an indication of the uncertainty of the final landing position with limited viewing duration of the trajectory. Using two starting positions of the ball, we have pairs of trials that require the same movement amplitude but have different sensory uncertainty due to different horizontal ball velocities (e.g., black ball falling straight down and gray ball being projected with 15 cm/s towards the subject). Similarly, we have trials where the same sensory uncertainty is traded off with two different motor uncertainties coming with different movement amplitudes, as shown in the thick black and gray lines. Here the ball is projected with the same horizontal speed, but due to the different starting positions of the ball requires movements of different amplitude to catch the ball. **(C)** The tradeoff between sensory and motor uncertainty. Here we illustrate the tradeoff between sensory and motor uncertainties using two trials as examples. Sensory uncertainty is represented by the solid line and two motor uncertainties by the dashed lines. As the trial time progresses, sensory uncertainty decreases as more of the trajectory has been seen by the subject. By contrast, motor uncertainty increases as the trial progresses. Here we show two examples of motor uncertainty curves; the lower one represents a movement of small amplitude (e.g., for the ball falling along the gray path in **B**) and the upper one when the amplitude of the required movement is longer (e.g., for the ball falling along the black path in **B**). In each trial, the point where the sensory and motor uncertainty curves intersect is the optimal switching time. The sensory uncertainty at the optimal switching time is denoted as σ1_*sensory*_ and the motor uncertainty as σ1_*motor*_. The combined minimum is given by the equation 3 in section “Analysis”. At the intersection, we can see a valley like structure being formed. The circular arc indicates the valley angle (as calculated in equation 6). Here we show the contrast between two trials where the ball is projected with the same velocity but different starting position of the ball and hence different motor uncertainty curves. **(D)** Understanding the experiment variables. The colored surface reflects sensory motor uncertainty, which depends on the required movement amplitude and trial time. The figure illustrates the uncertainty surface when the ball starts from the top right (shown here in black). An amplitude of 30 cm (top of the figure) corresponds to greater ball velocity and the bottom of the curve (around 10 cm) has the lowest ball velocities. The situation is reversed when the ball starts from the left where the gray ball is drawn. The white curve highlights the minimum sensory motor uncertainty, thereby defining the optimal switching times. The black dots are experimentally observed switching times. To relate variability in switching times to properties of the sensory motor uncertainty surface, we divided the entire velocity/amplitude range into bins with velocity range of 1.5 cm/s, shown here as a rectangle. The variability in switching time is calculated as the standard deviation of the observed switching times that lie within the bin/rectangle. The color of the heat map here indicates the combined sensory-motor uncertainty, calculated by combining σ1_*sensory*_ and σ1_*motor*_ in **(C)** using equation 3. The shallowness of the valley is the steepness with which sensory motor uncertainty increases when moving away from the optimal switching time, which can be estimated from this figure as the region with the same color. **(E)** Relationship between sensory-motor uncertainty and shallowness of the valley for balls starting from the left (filled dots) and right (open dots), in a control subject. Each dot in **(E)** is computed from a single bin (example rectangle shown in **D**). We divide the entire velocity range in 10 equal bins and compute the average predicted minimal sensory-motor uncertainty σ_*average*_ (equation 5) and the shallowness of the valley θ_*average*_ (equation 6). **(F)** Same as **(E)**, but for a patient.

We used a modified version of a virtual ball catching experiment [first reported by Faisal and Wolpert (2009) and modified in our earlier work Sengupta et al. (2018)]. Each experiment session consisted of three tasks: a sensory task to estimate sensory uncertainty, a motor task to estimate motor uncertainty and a combined task to test the tradeoff between sensory processing and motor execution. The conceptual design of the experiment, stimulus design, and experiment parameters are summarized in Figures 1, 2, and in Section “Experiment parameters.” Briefly, in the combined task subjects were instructed to catch a ball falling in a parabolic trajectory. Critical to the experiment, the ball disappeared as soon as subjects initiated their paddle movement to catch the ball. The disappearance of the ball on movement initiation split each trial into two distinct phases – a sensing phase and an acting phase. Therefore, the time of movement initiation will be referred to as *switching time*. The key idea is that the subjects switch from sensing to acting such that they maximize the probability of catching the ball. To maximize the probability of catching the ball, an ideal subject would minimize the expected absolute distance between the paddle and the ball, when it lands on the ground. The variability in the end-point of the paddle is the combined effect of sensory uncertainty about the landing position of the ball and uncertainty introduced by variability in the motor execution. In order to make predictions about optimal switching times, we quantified the sensory and motor uncertainty in two separate tasks.

The sensory task was designed to estimate sensory uncertainty as a function of the viewing duration of the ball and its horizontal velocity. As in the combined task, the ball falls in a parabolic trajectory. However, unlike the combined task, the time during which the ball was visible was pre-determined and subjects were given infinite time to indicate where the ball would have landed. For each combination of ball velocity and sensing time, the standard deviation of the error between true and estimated landing location was used to quantify sensory uncertainty. Sensory uncertainty was modeled as a multiplicative model with the horizontal velocity of the ball and the sensing time.

Similarly, the motor task was used to estimate motor uncertainty as a function of movement duration and movement amplitude. In each trial, the movement duration and amplitude were predetermined. The standard deviation of error between reach endpoints and target location was used to quantify motor uncertainty, for each combination of movement time and movement amplitude. Motor uncertainty was fitted using a reformulation of Fitts’ law, which modeled motor uncertainty as a function of movement time and movement amplitude (see below for formula). The rationale for the experiment was that the combined sensory-motor uncertainty could be estimated from the individual sensory and motor uncertainties. Using the estimated sensory-motor uncertainty, we could predict the optimal switching time such that the combined sensory-motor uncertainty was minimized (see section “Analysis” on analysis for the explicit formulation).

Using the rationale outlined above, we predicted the optimal switching times for both the PD and control groups. The aim of the current paper was to test (1) whether or not PD patients switch earlier than the controls, and (2) whether PD patients switch from sensing to acting such that the combined sensory-motor uncertainty is minimized, that is, if PD patients choose optimal switching times.

### Procedure

Data were collected in two sessions. In one session all ball trajectories would start from the top left corner, in the other from the top right corner. The stimuli used in the experiment are illustrated in Figure 1. Five subjects in each group started with the left start position and the other five started with the right start position. Before the start of each session, subjects were given detailed verbal and written instructions. In addition, subjects were given short practice sessions to familiarize themselves with the set-up and task requirements. The time between sessions was maximally 14 days. In each session, subjects performed all the three tasks: sensory, motor and combined task. Every task began with a practice block of 50 trials; all participants were sufficiently familiar after completion of at most 30 trials. A mandatory break was enforced after every 100 trials. On average, a session lasted 2.5–3 h, including the breaks.

### Experiment parameters

The experimental stimuli were drawn against a black background. Across the three tasks, ground level was shown as a 30 cm long white line, aligned with the movement axis of the paddle. The starting position of the paddle was always 5 cm to the right of this line, 20 cm to the right of the subjects’ midline. In both the sensory and combined task, simulated ball (green in color with a radius of 0.5 cm) trajectories started from the top left or top right corner of the screen, 15 cm from the body midline and 25 cm above ground level. The ball moved in a parabolic trajectory under simulated gravity (*g* = 25.5 cm/s^2^) and with an initial downward velocity of 0 cm/s, resulting in a flight time of 1.4 s. Horizontal velocity was selected in a pseudorandom order from 0, 2, 4, 6, 8, 10, 12, 14 cm/s for the sensory task (Figure 1A) and from a uniform distribution over the 0–15 cm/s interval in the combined task (Figure 1C). Balls starting on the left moved right, balls starting from the right moved left. The balls always landed on the white line indicating the ground level and to the left of the paddle. The development of the ball trajectory for the left starting position and two different velocities are illustrated in Figure 2A.

In the sensory task, the ball was visible for a predetermined sensing time which was chosen in a pseudorandom order from 200, 400, 600, 750, and 950 ms. The choice of ball velocity and sensing time pairs was made to ensure that the entire range of sensing times and associated sensory uncertainties could be estimated. Every pair of ball velocity and sensing time was tested 11 times, making a total of 440 trials for each ball starting position. The ball fell along with a horizontal bar. The bar continued to fall even after the ball had disappeared. The falling bar functioned as a timing aid. The distance between the falling bar and the ground line at any point in the trial provided subjects with an estimate of remaining time. At 1400 ms the bar reached the ground level. The paddle was clamped to the start position by the manipulandum until the ball had vanished (but the bar was still falling). After disappearance of the ball, subjects had infinite time to bring the paddle (red square, 0.5 cm wide) to the estimated landing position. Subjects pressed the space bar of a keyboard with their left hand to finish the trial. At the end of the trial subjects were shown the actual landing position as feedback.

In the motor task (Figure 1B), the reach target was shown (green square, 0.5 cm wide) on the white line that defined ground level. Targets appeared pseudo randomly 10.2, 12.3, 15.8, 17.9, 21.4, 26.3, and 29.8 cm left of the paddle’s start position. The required movement time was indicated to the subject by showing a horizontal bar falling from the top of the screen, starting 25 cm above ground level, at a constant velocity till it reached ground level. Required movement times were drawn pseudo randomly from 450, 650, 800, 1000, and 1200 ms. Every target position and movement time pair was tested 14 times, making a total of 560 trials across the entire experiment. These 560 trials were divided in two halves of 280 trials between the two experiment sessions. That is on each session, subjects performed seven repetitions of each target position and movement time pair. Subjects were instructed to make accurate movements to the target in the given time. Subjects could only start their movement after they had seen the bar falling from the top of the screen to indicate required movement time. Subsequently the subject initiated paddle movement (speed > 2.5 cm/s) and the bar would begin to fall again to keep track of the elapsed time. The end point of the movement was defined as the position of the paddle the moment the required movement time had expired, irrespective of the velocity of the hand. The actual and required endpoint of the reach were shown to the subject. The error on each trial was computed as the distance between the center of the paddle and the center of the target.

The sensory and the motor tasks test the two phases of the combined task. The key aspect of the combined task is that subjects decide when the sensory phase ends and the motor phase starts. In the combined task (Figure 1C), the falling ball disappeared as soon as the subject moved the paddle (speed > 2.5 cm/s), but the moving bar continued to fall to function as a timer. Subjects were instructed to catch as many balls as possible. To keep the subjects engaged, four different paddle widths were used (selected in a pseudorandom order 0.5, 1, 2, 4 cm). After every trial visual feedback was provided about the ball’s landing position and the position of the paddle when the ball reached the ground level. If the ball landed on the paddle, that is the distance between the center of the ball and the center of the paddle at the end of the trial was less than half of the paddle width, the trial was successful. Every 100 trials the number of catches was shown to the subject. In each session, subjects performed 400 trials of the combined task. Starting position of the ball did not change during the session.

Across the two sessions, each subject had to complete 2240 trials (800 (combined task) + 880 (sensory task) + 560 (motor task)). In the sensory task, each combination of ball speed (8) and sensing time (5) along with the starting position of the ball (2) was tested 11 times. In the motor task each combination of movement amplitude (8) and movement time (5) was tested 14 times. The number of trials per condition in the motor task was chosen to be slightly greater than sensory task to cover for the possibility that patients were not able to make high speed movements in a few trials. By measuring 14 trials of each type, even after excluding the improper trials we still could have enough trials for a reliable estimate of sensory and motor uncertainties. As mentioned in section “Paradigm,” we use a modified version of the ball catching task. In our earlier work we tested and validated that the modifications introduced were still able to capture near optimal sensory-motor tradeoff. In addition, our modifications allowed us to investigate the variability observed in switching times, Briefly, the two starting positions allow us to test the behavior in trials which require movements of same amplitude but with different uncertainty (illustrated in Figures 2B,C). This design aspect is further emphasized in section “Analysis” where we outline the analysis methods and section “Optimal switching times,” where we report our finding on the variability in switching time.

### Analysis

In the sensory uncertainty experiment, for each starting position of the ball, every combination of ball velocity and sensing time was repeated 11 times. The distribution of the indicated landing positions was used to estimate the subject specific parameters of the sensory uncertainty model. The sensory uncertainty was modeled as a multiplicative model with the horizontal velocity (*v*) of the ball and the sensing time (*st*).


(1) $$\sigma_{s\,e\,n\,s\,o\,r\,y}=a\cdot s\,t^{b}\cdot v^{c}$$

The free parameters of the model were fitted using maximum likelihood estimation. We computed the log-likelihood of the indicated landing positions being drawn from a normal distribution with mean at the true landing position and the standard deviation given by the model.

Similarly, the parameters for the motor uncertainty model were estimated based on the discrepancy between the endpoint of the reach and the required endpoint. Every combination of movement time and movement amplitude was repeated 14 times. The motor uncertainty was modeled using a reformulation of Fitts’ law, describing motor uncertainty as a function of movement time (*mt*) and movement amplitude (*x*).


(2) $$\sigma_{m\,o\,t\,o\,r}=x\cdot 2^{\left(1-\frac{m\,t-d}{e}\right)}$$

Under the assumption that the sensory and motor uncertainty are mutually independent the combined uncertainty, for a given switching time, can be estimated from the sensory and motor uncertainties:


(3) $$\sigma_{c\,o\,m\,b\,i\,n\,e\,d}^{2}=\sigma_{s\,e\,n\,s\,o\,r\,y}^{2}+\sigma_{m\,o\,t\,o\,r}^{2}$$

The duration of the trial (denoted as *T*) was always 1.4 s. That is sensing time (*st*) in equation 1 is at the expense of movement time (*mt*) in equation 2. In other words, if the subject switches after observing the ball for *t* seconds, there is *T-t* left for executing the movement. Thus, the combined sensory-motor uncertainty for a given switching time *t* becomes:


(4) $$\sigma_{c\,o\,m\,b\,i\,n\,e\,d}^{2}\,\left(t|v,x\right)={\left(a\cdot t^{b}\cdot v^{c}\right)}^{2}+{\left(x\cdot 2^{1-\frac{T-t-d}{e}}\right)}^{2}$$

Using equation 4 and the parameters capturing sensory and motor uncertainty, we can estimate the combined uncertainty as a function of time on any trial in the combined task, given that horizontal ball velocity (v) and the required movement amplitude (*x)* are known. Theoretically, a subject could choose any switching time between zero and *T*. However, an ideal performer would choose *t*_*optimal*_ such that the σ_*combined*_ is minimized. We refer to *t*_*optimal*_ as the optimal switching time.

Within the sensory-motor tradeoff framework (using equation 4), we can only compute the optimal switching time, i.e., the time point at which an ideal observer would start acting, but does not explain variability around the optimal switching time. In our earlier work (Sengupta et al., 2018) we found that the variability in switching time correlated with parameters derived from the optimal sensory-motor noise tradeoff framework (equations 1–4). We follow the same approach here and correlate the observed variability in switching time with the predicted minimal sensory-motor uncertainty σ_*combined*_(*t*_*optimal*_) on each trial. This minimal sensory-motor uncertainty defines the likelihood of end positions of the paddle and as such is also an estimate of the probability of success. Furthermore, we correlated the shallowness of the valley (see Figure 2C) around the optimal switching time with the observed variability in switching time. Shallowness of the valley provides an estimate of the sensitivity of task outcome to small variations in switching time. That is when the sensory and motor uncertainty curves decrease sharply and plateau, we have a wide minimum valley. As a result, large variations in switching time result in minor modulation in the uncertainty of the task outcome.

To compute the variability in switching time we divide the observed switching times into bins based on initial velocity and starting position of the ball (see Figure 2D). We divided the range of initial velocities into 10 bins with a range of 1.5 cm/s (corresponding to 2.1 cm amplitude). Each bin consisted of ∼40 trials. The variability in switching time was calculated as the standard deviation of the switching times within each bin. Similarly, for every bin, we computed the average predicted minimal sensory-motor uncertainty using:


(5) $$\sigma_{a\,v\,e\,r\,a\,g\,e}=\frac{1}{t\,r\,i\,a\,l\,s}\cdot \sum_{t\,r\,a\,i\,l\,s}\sigma_{c\,o\,m\,b\,i\,n\,e\,d}\,\left(t_{o\,p\,t\,i\,m\,a\,l}\right)$$

Next, we calculated the average shallowness of the valley within the bins. The shallowness of the valley was defined as the angle between the sensory and motor uncertainty curves at *t*_*optimal*_. The angle was calculated using:


(6) $$\theta =a\,r\,c\,t\,a\,n\,\left[\frac{\left(s-m\right)}{\left(1-s\cdot m\right)}\right]$$

where *s* is the slope of the tangent to sensory uncertainty curve and *m* is tangent to motor uncertainty curve at *t*_*optimal*_. The angles were averaged across the trials in each bin to calculate θ_*average*_. Next, we calculated the Pearson’s correlation between the experimentally observed variability in switching time and the predicted minimal sensory-motor uncertainty (σ_*average*_) and shallowness of the valley (θ_*average*_).

Finally, we used a combined regression model to predict the variability in switching time.


(7) $$\sigma_{s\,t}=\beta_{0}+\beta_{1}\cdot \theta_{a\,v\,e\,r\,a\,g\,e}+\beta_{2}\cdot \sigma_{a\,v\,e\,r\,a\,g\,e}$$

Note that for the two starting positions of the ball, θ_*average*_ and σ_*average*_ were correlated in different ways (see Figures 2E,F). When the ball started from the left, θ_*average*_ was an increasing function of σ_*average*_, whereas if the ball started from the right θ_*average*_was a decreasing function of σ_*average*_. This means that if we did not use two starting positions of the ball, θ_*average*_ will always be co-linear with σ_*average*_. Since we use two stating positions, we analyze the data from the two conditions together in a regression model. Pooling the data from both the conditions together θ_*average*_ and σ_*average*_ are no longer co-linear. This allowed us to tease apart the individual contributions of σ_*average*_ and θ_*average*_ to switching time variability.

All analysis was done using MATLAB 2015b. Unless otherwise stated the alpha value is set to 0.05.

In summary, we have multiple behavioral outcome measures and multiple model derived outcome measures. In Table 1 we summarize these measures, in addition to the detailed descriptions above.

**TABLE 1:** Summary of behavioral and model derived measures.

Switching time	Transition point from sensing to acting, i.e., disappearance of the falling ball at movement onset.
Optimal switching time	Maximizes catch performance, based on independent quantification of sensory and motor uncertainty.
Sensory uncertainty	Uncertainty about landing position, which depends on horizontal ball velocity and viewing duration.
Motor uncertainty	Uncertainty in the final paddle position, which depends on movement duration and movement amplitude.
Minimal sensory-motor uncertainty	The combined uncertainty at the optimal switching time.
Shallowness of the valley	Measure of the rate of change of sensory-motor uncertainty around its minimum at the optimal switching time.
Switching time variability	The fluctuations in switching time for a given range of landing positions.
Correlation between switching time variability and minimal sensory-motor uncertainty	Measure of exploratory behavior to improve catching performance.
Correlation between switching time variability and shallowness of the valley	Measure of subject’s knowledge of the effect variability in switching time has on task performance.

### Exclusion criteria for trials

No trials were excluded from the combined task. In both the sensory and motor uncertainty task, trials where the errors were uncharacteristically large were removed. The outliers were identified for each experimental condition, i.e., a specific combination of sensing time and ball velocity in the sensory condition or a specific combination of required movement time and amplitude in the motor task. All trials where the end point of the movement was not within 2.5 standard deviations of the mean endpoint were excluded from further analysis. This resulted in less than 4% of trials removed from the sensory uncertainty data. In the motor uncertainty experiment, some subjects could not reach the farthest targets in the shortest movement times. The experimental conditions (movement time and amplitude pair) where the correct endpoint was not within 2.5 positive standard deviations of the average endpoint were identified as undershoot cases. The entire condition was then excluded from further analysis. Thus, trials in the motor task were removed if they met either the outlier criterion (described above) or the undershoot criterion. This resulted in 0.5% of the trials (348) removed from further analyses, of which 42 trials because they undershot the target systematically.

In the PD group, subject 7 completed only half of the motor uncertainty experiment (280 trials instead of 560 trials). Therefore, data from subject 7 was not included in any statistical comparisons and supporting figures. However, the data is provided and highlighted in all the tables.

## Results

The aim of the study was to test whether PD patients’ switch earlier than controls and if such a tendency results in suboptimal sensory sampling and compromised catching performance, which would be a sign of disinhibition, or that behavior is still statistically optimal given their sensory and motor uncertainty. To this end, our subjects executed three variants of a virtual ball catching task. One for estimating their sensory uncertainty, one for estimating their motor uncertainty and a combined task for testing whether they optimally tradeoff sensory and motor uncertainty.

### Sensory task

**FIGURE 3:**
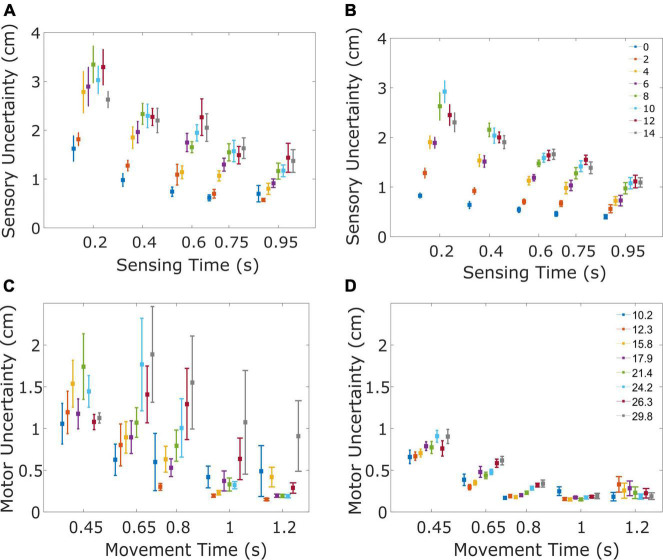
Sensory and motor uncertainty. The four panels illustrate the sensory **(A,B)** and motor uncertainty **(C,D)** for patients **(A,C)** and controls **(B,D)**. **(A)** Sensory uncertainty for the PD patients decreases with sensing time and increases with the speed of the ball. Sensory uncertainty is shown for the five tested sensing times, separated for the eight horizontal velocities of the ball (color of the marker, ranging from 0 to 14 cm/s). Markers indicate the mean uncertainty across subjects, error bars indicate the standard error. To ensure each data point is clearly visible, a small displacement has been introduced on the time axis. **(B)** Same as **(A)**, now for the healthy control subjects. **(C)** Motor uncertainty for the PD patients decreases with movement time and increases with the speed of the ball/amplitude of the movement. Motor uncertainty is shown for the five tested movement times, separated for the eight movement amplitudes. Markers indicate the mean uncertainty across the subjects and the error bar indicates the standard error. **(D)** Same as **(C)**, now for the healthy control subjects.

The sensory task served to estimate sensory uncertainty as a function of time spent observing the ball (*sensing time*) and the speed of the ball in the horizontal direction (*ball speed*). Pooled across all trials and subjects, patients missed the target by 0.19 cm and the standard deviation of the error distribution was 2.4 cm. The control group missed the actual landing position by 0.02 cm and the standard deviation of the error was 2.0 cm. Figures 3A,B show that the sensory uncertainty decreases with sensing time and plateaus for the longest sensing times, for both groups. Furthermore, for the same sensing time, the sensory uncertainty increases as the ball speed increases.

We summarized the data in Figures 3A,B by fitting a model with three subject-specific parameters (*a, b*, and *c*, see section “Materials and methods”) which quantify the effects of baseline uncertainty, sensing time and ball speed respectively. We verified that the parameters did not differ between the two starting positions of the ball (see Table 2 for the parameters and statistical comparisons). For further analyses we pooled the data across both conditions. Sensory uncertainty predicted by the model (using parameters from the pooled analysis) correlated with the observed sensory uncertainty with an average Pearson correlation coefficient of 0.76 (range PD: 0.40–0.74; Control: 0.70–0.88). The parameter *b* was negative for all subjects, with an average of –0.63 (*SD* = 0.21), confirming that sensory uncertainty decreases with sensing time. The value of *b* was slightly lower for the patient group (average = –0.68, *SD* = 0.21) compared to the control group (average = –0.58, *SD* = 0.21), but the difference was not statistically significant [*t*(17) = –1.04, *p* = 0.31]. The parameter *c*, quantifying the effect of ball speed, was positive for all subjects with an average value of 0.34 (*SD* = 0.08). The value of *c* was slightly smaller for the PD group (average = 0.30, *SD* = 0.08) compared to the control group (average = 0.37, *SD* = 0.08). However, the difference was not statistically significant [*t*(17) = –1.67, *p* = 0.11]. The parameter *a*, which quantified baseline uncertainty, also did not differ between the groups [*t*(17) = 1.33, *p* = 0.20]. In summary, there is no statistical difference in the parameters quantifying sensory uncertainty between the PD-group and the controls.

**TABLE 2:** Parameters for the sensory uncertainty model (equation 1), computed separately for the two starting positions of the ball.

Healthy controls						
	Parameter *a*		Parameter *b*		Parameter *c*	
	Left	Right	Left	Right	Left	Right
C1	0.40	0.53	–0.60	–0.45	0.52	0.44
C2	0.68	0.67	–0.33	–0.29	0.32	0.29
C3	0.51	0.47	–0.48	–0.50	0.41	0.37
C4	0.25	0.27	–0.88	–0.78	0.41	0.45
C5	0.33	0.37	–0.77	–0.83	0.30	0.33
C6	0.32	0.39	–0.41	–0.52	0.45	0.25
C7	0.62	0.36	–0.20	–0.54	0.43	0.41
C8	0.48	0.39	–0.45	–0.52	0.39	0.31
C9	0.53	0.41	–0.82	–0.58	0.18	0.34
C10	0.41	0.25	–0.84	–0.93	0.45	0.41
	*t*(9) = 1.17, *p* = 0.27		*t*(9) = 0.35, *p* = 0.73		*t*(9) = 0.86, *p* = 0.41	

**PD patients**						
P1	0.28	0.28	–0.97	–0.76	0.34	0.38
P2	0.31	0.50	–0.89	–0.56	0.52	0.43
P3	0.94	1.04	–0.23	–0.27	0.37	0.21
P4	0.45	0.62	–0.97	–0.85	0.37	0.33
P5	0.31	0.33	–0.96	–0.91	0.36	0.25
P6	0.53	0.48	–0.72	–0.85	0.20	0.19
*P7*	*4.34*	*4.99*	–*0.24*	*0.08*	*0.02*	*0.13*
P8	0.47	0.31	–0.59	–0.59	0.36	0.42
P9	0.95	0.62	–0.35	–0.65	0.18	0.19
P10	0.80	0.56	–0.60	–0.97	0.29	0.28
	*t*(8) = 0.55, *p* = 0.59		*t*(8) = 0.18, *p* = 0.86		*t*(8) = 1.50, *p* = 0.17	

Subject P7 was not included in the statistical comparisons and is presented in italics.

### Motor task

The aim of the motor task was to quantify motor uncertainty as a function of movement time and movement amplitude. In the motor task patients missed the target by 0.28 cm and the standard deviation of the error was 1.04 cm. The control subjects missed the target by 0.12 cm while standard deviation of the error was 0.5 cm. Figures 3C,D show that motor uncertainty decreases as movement time increases. Furthermore, for the same movement time, motor uncertainty increases with movement amplitude. Motor uncertainty was modeled using two subject-specific parameters *d* and *e* (see section “Materials and methods”). Parameter *e* quantifies the movement time required for the motor uncertainty to plateau. The ratio *d/e* captures the maximum motor uncertainty for the shortest movement times. Motor uncertainty predicted by the model correlated with the observed motor uncertainty with an average Pearson correlation coefficient of 0.80 (range PD: 0.56–0.95; Control: 0.39–0.93). Parameter *e* did not differ between groups [*t*(17) = –1.78, *p* = 0.09, average for patients was 0.27 (*SD* = 0.08), average for controls was 0.32 (*SD* = 0.06)] indicating that for longer movement durations the motor uncertainty did not differ between both groups. Parameter *d* was less negative for patients [*t*(17) = 2.63; *p* = 0.02, average for controls: –1.37 (*SD* = 0.35), average for patients: –0.77 (*SD* = 0.56)]. This also means that the ratio *d/e* was larger for patients [*t*(17) = –2.99, *p* < 0.05], indicating that for shorter movement times their motor uncertainty was higher. In summary, motor uncertainty is significantly greater in the PD-group, especially for faster movements.

### Combined task

#### Catch performance and observed switching times

On average, PD patients caught 47% balls (range 34–72%), whereas the control group caught 64% of the balls (range 49–76%). This difference failed statistical significance [*t*(17) = –1.97, *p* = 0.07].

The average switching time for the PD group was 603 ms (range 526–680 ms) and for the HC group was 636 ms (across all trials, range 503–775 ms) and did not differ significantly [33 ms, *t*(17) = –1.16; *p* = 0.265].

A notable feature of PD patients’ performance was that they were still moving at the time of the ball landing, i.e., 1.4 s, as indicated by the falling bar, acting as timer. Given the timing information marking the end of the trial, the hand position at 1.4 s was the position used for analysis and considered the patients’ intended catch response.

The greater velocity of patients, at interception, did not involve a systematic tendency to overshoot the target. The proportion of undershoot to overshoot trials was 46/54 for controls and 42/58 for patients. Likewise, the velocity at the end of the trial was significantly slower for undershoot trials than for overshoot trials, both for controls and patients. Overshoot was far more likely to occur when the ball started from the right, and undershoot was more likely with balls starting left. Again this pattern was the case in patients and controls alike. Based on these results it seems fair to take the hand position at 1.4 s as the position patients aimed for to catch the ball.

#### Optimal switching times

**FIGURE 4:**
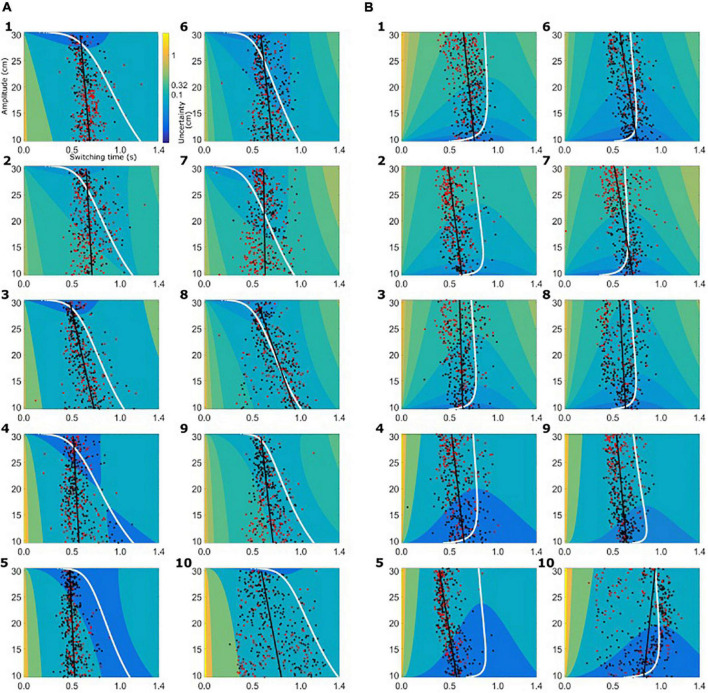
Sensory-motor uncertainty and switching times for controls. For every subject the sensory-motor uncertainty map is shown as a function of movement amplitude (vertical axis) and viewing duration (horizontal axis). Warmer colors indicate greater value for combined sensory-motor uncertainty. For each amplitude, the combined sensory-motor uncertainty is high at the beginning of the trial and reduces in time, reaching the smallest value somewhere in the middle of the trial duration. The white curves indicate the time at which the combined sensory-motor uncertainty is at its minimum, and thus switching from sensing to acting would be optimal. The circular markers indicate the observed switching time. Black markers indicate successful catches, red markers unsuccessful catches. The black line represents the linear regression of the observed switching time with the amplitude. **(A)** Data from healthy controls for the condition where the ball starts from the left. **(B)** Data from the healthy controls for the condition where the ball starts from the right.

**FIGURE 5:**
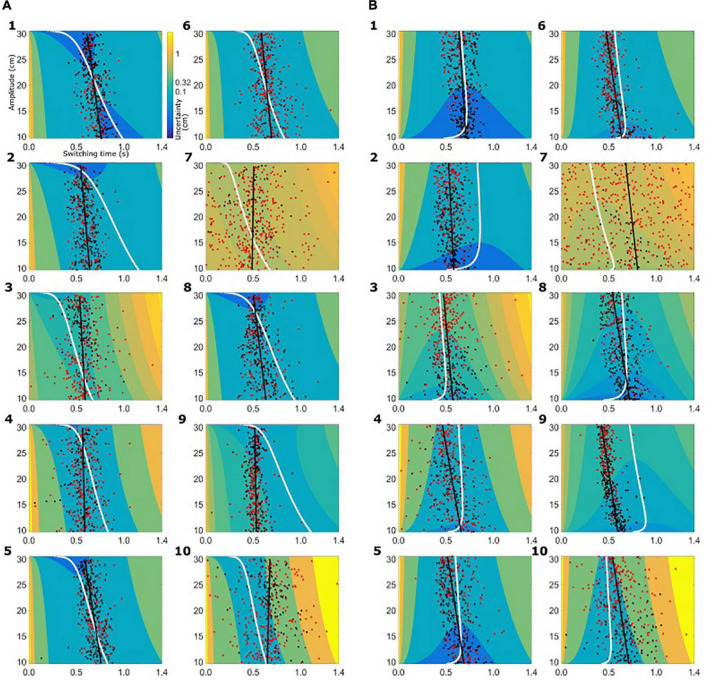
Sensory-motor uncertainty and switching times for patients. The figure follows the same set-up as Figure 4. **(A)** Data from patients for the condition where the ball starts from the left. **(B)** Data from patients for the condition where the ball starts from the right.

Based on the subject-specific sensory and motor uncertainty, we computed the theoretical uncertainty in the combined task (σ_*combined*_, see equations 3 and 4) as a function of movement initiation time. As the trial proceeds, sensory uncertainty decreases and motor uncertainty increases. So, early in the trial combined uncertainty was high, driven by the sensory uncertainty, and toward the end of the trial the combined uncertainty was high again, but now dominated by motor uncertainty. Therefore, if we plot the combined uncertainty, σ_*combined*_, as a heat map with trial time on the horizontal axis and movement amplitude on the vertical axis, we see a clear valley of low combined uncertainty, flanked on both ends of the time axis with higher values of uncertainty. Figure 4 shows the uncertainty maps for all healthy controls and Figure 5 shows the uncertainty maps for patients. Data for balls starting from left are plotted in Figure 5A and data for the trials where ball starts from the left are plotted in Figure 5B. For each time point during the trial, combined uncertainty increases as the velocity of the ball increases. That is, in Figures 4A, 7A, for each time point the uncertainty decreases as the amplitude increases. This was because if the ball started from the left, larger velocity resulted in smaller amplitude of movement as the ball was approaching the starting position of the subject. This reversed when the ball started from the right. Higher ball velocity resulted in greater amplitude of movement. Hence in Figures 4B, 5B we see that for a fixed time point during the trial, uncertainty increased as the amplitude increased. The white curve, through the sensory-motor uncertainty map, highlights the time at which the combined uncertainty was at its minimum, i.e. the ideal switching time. The observed switching times of the individual trials are indicated by the dots in Figures 4, 5. For trials where the subject caught the ball, the dot is colored black and trials where the subjects missed the ball are shown in red.

**FIGURE 6:**
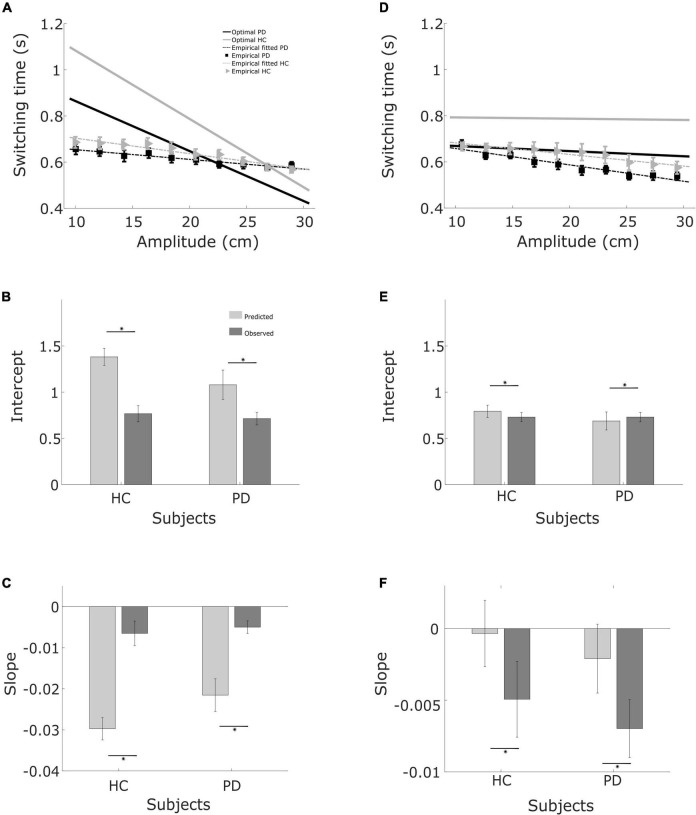
Regression fits and bar graphs with the fit coefficients. **(A)** Relationship between required movement amplitude and switching time. Linear regression curves and optimal curves for balls starting from the left, in both PD patients and controls. **(B)** Intercept for balls starting from the left for controls and patients. **(C)** Slope for balls starting from the left for patients and controls. **(D)** Same as **(A)**, but for balls starting from the right. **(E)** Same as **(B)** for balls starting from the right. **(F)** Same as **(C)**, for balls starting from the right.

**FIGURE 7:**
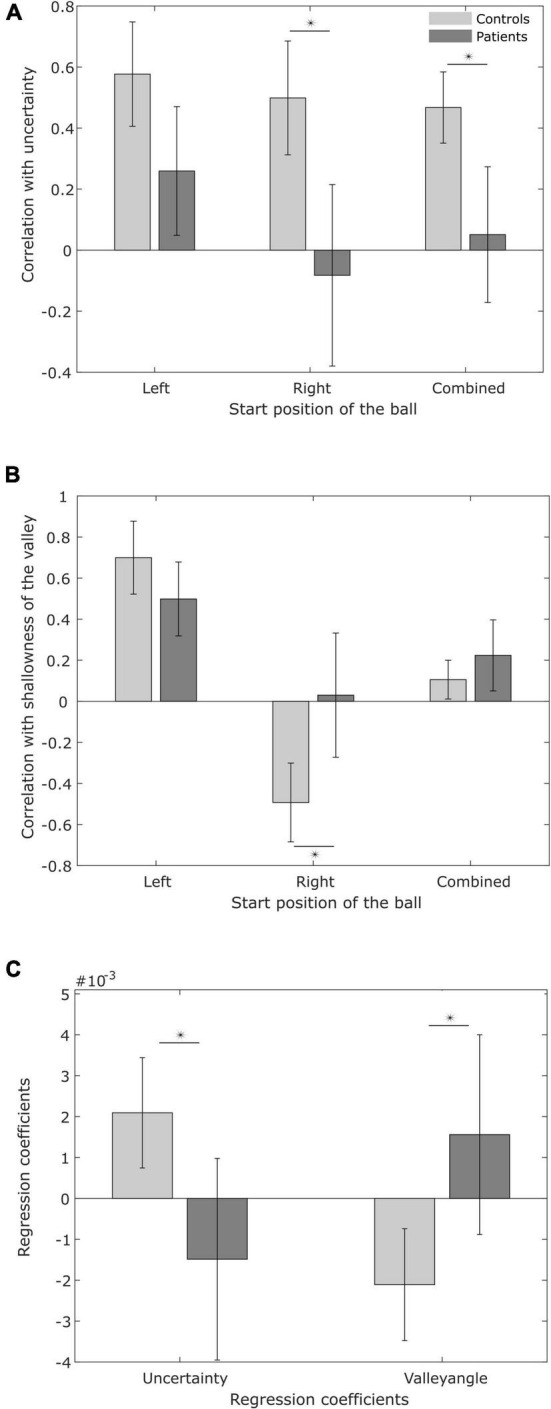
Variability in switching time. **(A)** Pearson correlation between the variability in switching time and predicted minimal sensory-motor uncertainty, for patients and controls. **(B)** Pearson correlation between the variability in switching time and shallowness of the valley. **(C)** Regression coefficients for predicted minimal sensory-motor uncertainty and shallowness of the valley as explanatory variables for observed switching time variability.

The switching time was dependent on the velocity of the ball and the required movement amplitude to catch the ball. We approximated this dependence of observed switching time as a linear function of required movement amplitude, shown as the black solid line in the plots of Figures 4, 5. In the individual plots of Figures 4, 5 we see that both the observed switching times for patients and controls deviated from the predicted (optimal) switching time (white line). Indeed, we found that the fit parameters for the predicted switching time were significantly different from the fit parameters for the observed switching time in Figures 4, 5 (regression parameters for the individual subjects can be seen in Table 3). The difference between the observed switching times for the two conditions – balls starting from the left and right – was not significant. Details of the statistical comparisons between the regression parameters are summarized in Supplementary Table 1.

**TABLE 3:** Linear regression between switching time and required movement amplitude for the individual subjects.

Balls from left							
Intercept				Slope			
HC		PD		HC		PD	
Predicted	Observed	Predicted	Observed	Predicted	Observed	Predicted	Observed
1.54	0.74	1.23	0.82	–0.0337	–0.0047	–0.0269	–0.0068
1.41	0.74	1.44	0.68	–0.0301	–0.0027	–0.0302	–0.0042
1.32	0.85	0.84	0.64	–0.0291	–0.0121	–0.0189	–0.0035
1.46	0.67	0.99	0.63	–0.0347	–0.0056	–0.0169	–0.0023
1.34	0.59	0.99	0.82	–0.0249	–0.0037	–0.0183	–0.0075
1.24	0.76	0.99	0.78	–0.0263	–0.0055	–0.0185	–0.0064
1.32	0.65	*0.83*	*0.46*	–0.0314	–0.0013	–*0.02*	*0.0043*
1.26	1.01	1.15	0.71	–0.0275	–0.0147	–0.0251	–0.0068
1.29	0.77	1.32	0.56	–0.0249	–0.0056	–0.026	–0.0015
1.64	0.88	0.76	0.8	–0.0347	–0.0093	–0.0133	–0.006

**Balls from right**							
0.78	0.82	0.71	0.75	0.0039	–0.0061	–0.0014	–0.0037
0.86	0.7	0.77	0.61	–0.003	–0.0064	0.0033	–0.0027
0.73	0.66	0.52	0.63	0.0009	–0.0016	–0.0027	–0.0049
0.69	0.71	0.66	0.68	0.0032	–0.0057	–0.0005	–0.0053
0.89	0.67	0.66	0.78	–0.0026	–0.0088	–0.0016	–0.0084
0.76	0.84	0.72	0.77	–0.002	–0.0086	–0.0051	–0.0102
0.67	0.78	*0.71*	*0.84*	0.0012	–0.0082	–*0.0138*	*0.0009*
0.73	0.66	0.65	0.82	–0.0011	–0.0028	–0.0003	–0.009
0.92	0.69	0.98	0.74	–0.0065	–0.0044	–0.0089	–0.0105
0.9	0.78	0.53	0.8	0.0025	0.0033	–0.0017	–0.0081

Analyses were done separately for the two starting positions of the ball and for predicted and observed switching times. Statistical comparisons on the parameters are summarized in Supplementary Table 1. Subject P7 is presented in italics.

Although the comparison of fit parameters shows a difference between the observed and predicted switching times, this does not entail a wide divergence or irrelevance of the optimal control analysis. To maximize catch performance, subjects should choose switching times such that the combined uncertainty is minimized. Hence we analyzed the combined sensory-motor uncertainty at the observed switching time [σ_*combined*_(*t*_*observed*_)] with reference to the predicted minimal sensory-motor uncertainty [σ_*combined*_(*t*_*optimal*_)]. We found that in 36% (median; range, 3–55%) of trials controls switched such that the combined uncertainty [σ_*combined*_(*t*_*observed*_)] was within 2.5% of the predicted minimal sensory-motor uncertainty. Moreover, in 71% (median; range, 11–87%) of trials controls switched such that the combined uncertainty was within 10% of the minimum achievable combined sensory-motor uncertainty. In the PD group, we found that 25% (median; range, 8–55%) of trials had switching times within 2.5% of the predicted minimal sensory-motor uncertainty and 72% (median; range, 28–87%) of trials were within 10% of the predicted minimal sensory-motor uncertainty.

When we summarize the data for both groups, the predicted optimal switching time, shown in Figure 6, is shorter for PD (solid black line) than for the healthy controls (solid gray line), irrespective of the starting position of the ball. This is primarily due to the greater motor uncertainty observed in the patients. Measured switching times deviated from the predictions in both patients and controls, and in the same direction. Two observations shed light on the difference, One is that predicted switching time (*t*_*optimal*_) when the ball starts from the right is markedly less influenced by movement amplitude. In the condition where balls start from the right, greater velocity of the ball requires a higher movement amplitude for catching the ball. Greater ball velocity and higher movement amplitude compete for a longer sensing time and longer time for acting, respectively. When the ball starts from the left, the situation is reversed. When the ball starts from the left with a greater horizontal velocity, the amplitude of movement required to catch the ball is shorter. Thus the tradeoff between sensory and motor phases is harsher when the ball starts from the right, attenuating the influence of movement amplitude.

The second observation is that the difference between patients and controls in observed switching times is considerably smaller than the difference predicted by the model (Figures 6A,D). Moreover, patients are closer to the optimal as compared to the controls. This is because the motor behavior captured in the motor uncertainty task indicates that controls can make fast and accurate movements. The model therefore predicts that it is optimal to allocate time to sensing to improve the estimate of the landing position and then use a fast movement to catch the ball. In practice, controls choose to move slower than their capacity. The same is also true for patients, however, patients use more of their movement capacity as compared to the controls.

#### Variability in switching time

In our previous work (Sengupta et al., 2018) we found that the variability in switching time was not entirely arbitrary, but was correlated with the shallowness of the valley and the predicted minimal sensory-motor uncertainty. The shallowness of the valley is a measure of how the predicted sensory-motor uncertainty changes in response to variability in switching time: the shallower the valley, the smaller the effect of variations in switching time on catch performance. The predicted minimal sensory-motor uncertainty is a measure of the probability of successfully catching the ball.

Earlier work on reach behavior has suggested an impairment in reward processing of PD- patients (Pekny et al., 2015). After an unrewarded trial, healthy controls increase their motor variability in subsequent trials, but this explorative behavior is compromised in PD-patients. We surmise that, because successful catch-performance is a measure of expected reward, explorative behavior in our task is linked to the minimal sensory-motor uncertainty. This leads to the hypothesis that PD-patients show smaller modulation of switching time variability with changes in sensory-motor uncertainty.

To examine this hypothesis, Figure 7A shows the average correlation between the predicted minimal sensory-motor uncertainty and switching time variability for both patients and controls, for the individual start positions of the ball and in combination. Table 4 presents the correlations at the subject level. While there is a significant positive correlation for the controls [combined: *t*(9) = 7.31, *p* < 0.001; left: *t*(9) = 6.16, *p* < 0.001; right: *t*(9) = 4.88, *p* < 0.001], this was not seen in the patient group [combined: *t*(8) = 0.44, *p* = 0.7; left: *t*(8) = 2.22, *p* = 0.06; right: *t*(8) = –0.28, *p* = 0.8]. The difference between controls and PD-patients supports the hypothesis on reduced explorative behavior in PD (Pekny et al., 2015) and suggests that patients’ behavior is less influenced by expected reward.

**TABLE 4:** Correlation between predicted minimum sensory-motor uncertainty and switching time variability, for healthy controls (HC) and PD patients, separated for the two starting locations of the ball.

Left		Right	
HC	PD	HC	PD
0.33	0.61	0.21	0.88
0.72	0.55	0.29	0.54
0.81	0.65	0.77	–0.12
0.35	0.39	0.26	–0.74
0.78	0.05	–0.05	–0.06
0.63	–0.17	0.47	–0.77
0.64	*0.06*	0.5	–*0.38*
0.83	0.61	0.96	0.14
–0.1	0.23	0.76	–0.03
0.77	–0.4	0.82	-0.28

Subject P7 is presented in italics.

We also examined the correlation between shallowness of the valley and switching time variability (Figure 7B). Qualitatively, with a shallow valley (or a wide blue region in Figures 4, 5), changes in switching time have only marginal effects on sensory-motor uncertainty, while a steep valley would yield large effects. Following this logic, one expects a positive correlation between shallowness of the valley and switching time variability.

For both controls and patients we do find a positive correlation for balls coming from the left [controls: *t*(9) = 7.20, *p* < 0.001; patients: *t*(8) = 5.67, *p* < 0.001]. However, for balls coming from the right, we found a negative correlation in the controls [*t*(9) = –4.69, *p* < 0.001]. The counterintuitive negative correlation is probably explained by the co-linearity of shallowness of the valley and minimal sensory-motor uncertainty with switching time variability (see Figures 2E,F). For the patients we found no correlation between switching time variability and shallowness of the valley when the ball started from the right [*t*(8) = 0.04, *p* = 0.97]. If we look at the correlation for the individual patients (Table 5), only four of the ten PD patients show a negative correlation and the others show a positive correlation. The positive correlation observed in the majority of the patients when the ball starts from the right may indicate that the PD patients utilize the freedom offered by the shallowness of the valley to not precisely control the switching time.

**TABLE 5:** Correlation between shallowness of the valley and switching time variability, for healthy controls (HC) and PD patients, separated for the two starting locations of the ball.

Left		Right	
HC	PD	HC	PD
–0.01	0.73	–0.17	–0.9
0.46	0.56	–0.22	–0.55
0.95	0.6	–0.75	0.21
0.9	0.42	–0.31	0.77
0.73	0.61	0.06	–0.11
0.48	0.79	–0.48	0.78
0.77	*0.07*	–0.49	*0.23*
0.95	0.85	–0.96	–0.21
0.89	0.49	–0.77	0.05
0.88	–0.13	–0.83	0.04

Subject P7 is presented in italics.

In order to tease apart the possible joint effects of minimum sensory-motor uncertainty and shallowness of the valley on switching time variability, we conducted a multiple regression analysis (see section “Materials and methods,” results are in Table 6). Figure 7C shows the regression coefficients associated with minimum sensory-motor uncertainty and shallowness of valley, for both patients and controls. For healthy controls the slopes were positive for both the predicted minimal sensory-motor uncertainty [*t*(9) = 4.75, *p* < 0.005] and the shallowness of the valley [*t*(9) = 5.51, *p* < 0.001]. This is in line with our earlier findings in young subjects (Sengupta et al., 2018). By contrast, in the PD group only the correlation between switching time and shallowness of the valley was positive [*t*(8) = 2.79, *p* < 0.05], while the correlation with minimal sensory-motor uncertainty was not significantly different from zero [*t*(8) = –0.38, *p* = 0.7]. Comparison between the groups showed that the regression coefficient for predicted minimal sensory-motor uncertainty was significantly larger for controls [*t*(17) = 3.40, *p* < 0.005] but that for shallowness of the valley did not differ between groups [*t*(17) = 0.54, *p* = 0.6]. Together this suggests that patients do not increase their switching time variability based on sensory-motor uncertainty, but only based on the shallowness of the valley.

**TABLE 6:** Regression coefficients for predicting switching time variability from both predicted minimal sensory-motor uncertainty and shallowness of the valley.

Intercept		Uncertainty		Shallowness	
PD	HC	PD	HC	PD	HC
−0.0083	–0.3517	0.0138	0.0980	0.0006	0.0022
−0.4143	–0.0028	0.0373	0.0169	0.0028	0.0004
−1.0660	–0.5842	0.0859	0.0096	0.0065	0.0036
−0.7267	–1.6081	0.0933	–0.0283	0.0050	0.0097
−1.1050	–0.7639	0.1200	0.0122	0.0068	0.0052
−0.2553	–1.2798	0.0506	–0.0504	0.0019	0.0090
*−0.3423*	0.3624	*0.0573*	–0.0076	*0.0023*	–0.0001
−1.0775	–0.9738	0.1608	0.0538	0.0063	0.0063
−1.0816	–0.3473	0.1779	0.0235	0.0064	0.0024
−1.4380	1.1263	0.2235	–0.0754	0.0092	–0.0037

Subject P7 is presented in italics.

To summarize, analyzing the correlation between variability in the switching times with predicted sensory-motor uncertainty and shallowness of the valley, suggests that PD patients are reluctant to start exploring when the predicted minimal sensory-motor uncertainty is high, but let go of precise control over switching time variability when the shallowness of the valley increases.

### Velocity profiles of the movements

In the combined task, we found that the PD group used an average peak velocity of 38 cm/s (*SD* = 4.40 cm/s) which was lower than the average peak velocity of 51 cm/s (*SD* = 6.35 cm/s) used by the HC group [*t*(17) = –5.00, *p* < 0.005]. However, at the moment the falling ball hit the floor, the PD group’s paddle still had an average speed of 4.92 cm/s (*SD* = 3.04 cm/s), which was greater than in the HC group [average = 1.71 cm/s, *SD* = 1.47 cm/s, *t*(17) = 2.97, *p* = 0.009]. If we consider the sum of viewing duration and actual movement duration (i.e. the time from the ball starting to fall to the hand velocity slowing below the threshold of 2.5 cm/s) as the trial duration, the mean trial duration for PD patients was 1.39 s, against 1.25 s for controls. That is, patients spent 138 ms longer than control subjects [*t*(17) = –4.67, *p* < 0.005] to catch each ball. This additional 138 ms can be regarded as devoted to the catching movement, making the proportion of the time devoted to viewing the ball shorter for PD patients compared to controls [average fraction devoted to sensing for PD was 0.47, for HC was 0.51, *t*(17) = 3.74, *p* < 0.005].

Not only patients, but also controls use greater peak velocities in the motor task compared to the combined task. Thus, for the ball landing positions comparable to the movement amplitudes tested in the motor task, subjects (patients and controls alike) chose switching times that allowed for longer movement times, requiring smaller peak velocities. This may be due to the motor task imparting a stronger sense of urgency compared to the more lenient time constraints of the combined task, which allowed for a tradeoff between sensing and acting. No less important is that participants aimed movements to a physically present target in the motor task, but towards an estimated location of the target in the combined task. The uncertainty about the target may have an effect on the kinematic parameters of the movement.

## Discussion

In this study, we explored how disinhibition in PD patients could manifest in a naturalistic task using a virtual ball-catching experiment and analyzing the data in a probabilistic optimal control framework. Key-element of the experiment was that initiation of the catching movement extinguished vision of the falling ball, separating the trial in a sensing and an acting stage. We found that PD patients did not switch significantly earlier than controls from sensing to acting. The computationally determined optimal switching times, derived from the individual estimates of sensory and motor uncertainties, deviated from the observed switching times for both groups, but did so in a similar way, with observed switching times for both groups mostly shorter than the predicted. Where PD patients did differ from the control group, was in the variability of the switching time. Patients showed reduced variability and a weaker correlation between variability in switching time and predicted minimal sensory-motor uncertainty. Analyses of the movement kinematics showed that patients still moved when they caught the ball. In control subjects’ catching movements, the timepoint at which the ball landed and the movement ended coincided. If we factor in the actual trial time that patients and controls used, we found that the patients spent a smaller proportion of the total trial time observing the ball. Here, we discuss the observed behavioral data, both within and outside the framework of optimal sensory-motor tradeoff.

### Switching time

First, let us discuss the between group comparisons of the raw behavioral data. There is an extensive literature on response slowing in PD in different types of reaction time tasks (e.g., Jahanshahi et al., 1992; Gauntlett-Gilbert and Brown, 1998; Wang et al., 1998). In most studies, PD patients are slower than controls, albeit not universally and not by a large margin. Paradoxically, in view of the characteristic bradykinesia in PD, reduced inhibition of prepotent response tendencies can even produce fast motor responses while generating response delays in conflict tasks. In line with this background, we found that, on average, patients switched 33 ms earlier than the controls. However, against the large variability (within the velocity conditions and between the subjects) the difference was not statistically significant.

We also found that the peak velocity that PD patients used in their catching movements was reduced compared to the controls, which concurs with the general slowness of movement observed in PD patients. However, their velocity at the end of the trial was greater than the velocity found in the controls. This observation suggests that PD patients and controls may be solving the sensory-motor tradeoff differently. Since PD patients were still moving at the end of the trial, they were using the entire 1.4 s of the trial whereas the controls were ending their trials 138 ms earlier. Thus, if we normalize the switching time by the actual trial duration, we find that PD patients do devote significantly less time to sensing. Note, however, that switching earlier is not necessarily detrimental to performance. Since motor uncertainty in PD patients was greater than in controls, early switching may in fact be more optimal for patients. This consideration underscores the rationale of the optimal control analysis.

As to why PD patients stopped moving at a later point in time than control subjects, one might ask whether this is related to impaired stopping in PD (e.g., Gauggel et al., 2004), hence a manifestation of disinhibition where we did not expect it. Although terminating a catching movement is very different from aborting an ongoing movement at a random cue, as in stop signal reaction time tasks, or controlling prepotent response tendencies, as in conflict tasks, we consider this the most likely explanation. Another consideration is that patients, while showing good performance when moving to a physically present target under a time constraint (e.g., Majsak et al., 1998; Fooken et al., 2022), are poorer when the target location is not given but is estimated, as in the combined task. Thus motor execution might differ depending on whether the target is physically present or not. One should also realize that movement planning already starts in the sensing stage and that planning during the limited sensing time perhaps evolved further in controls than in patients (in spite of similar performance in the sensing task), resulting in more accurate endpoint planning.

### Switching time and optimal control analyses

To put optimality in context of the ball catching task, the falling ball is the source of trajectory information and the longer the subject observes the falling ball the better they can estimate its landing position. The sensory information accumulates gradually, feeding into an upcoming motor plan (Selen et al., 2012; Brière and Proteau, 2017). The accumulation of sensory information can, even before analysis is complete, produce measurable activation of the motor cortex (Coles et al., 1985; Gratton et al., 1988; Praamstra et al., 1998; Selen et al., 2012). Therefore, an ability to suppress the accumulating sensory information, already fed forward to the motor cortex, from prematurely initiating movement is essential for observing the ball for an optimal amount of time.

Faster than normal movement initiation in PD is most frequently observed in reflexive saccades (Briand et al., 2001; Fielding et al., 2005; Chambers and Prescott, 2010; Duprez et al., 2017) but can also be seen with manual responses (Praamstra and Plat, 2001). These observations suggest hyperreflexive (visual) orienting in PD (Poliakoff et al., 2003). A related phenomenon is involuntary attentional capture by irrelevant distracters in visual search (Deijen et al., 2006). Importantly, Zhang et al. (2016) applied a hierarchical drift-diffusion model to the results of a saccadic go/nogo task. PD and PSP (progressive supranuclear palsy) patient groups exceeded controls in the number of commission errors, demonstrating impaired inhibition. The drift-diffusion analysis showed patient groups having shorter non-decision time but slower drift rate of accumulation, indicating a prepotency of responding in combination with a reduction in further accumulation of evidence (Zhang et al., 2016). Based on (i) the evidence for early activation of the motor cortex, and (ii) hyperreflexive visual orienting in PD, against the background of a large body of work on disinhibition in PD, our primary hypothesis was that impaired inhibition in PD patients would interfere with optimal sensory sampling related to sub-optimally early switching times.

The optimal control analyses predicted longer switching times than those actually produced. But this was the case for both groups, and there is no basis to claim the switching times were sub-optimally short, given that patients’ switching times were closer to optimal than those of controls and catch performance was not significantly different between groups. One might add that patients’ greater paddle velocity at the time of interception could be regarded as trading movement time for longer perceptual sampling. Hence, one may conclude that the tradeoff between sensory sampling and motor response initiation in patients is not compromised by premature movement initiation.

An important question is why the observed switching times deviated considerably from the predicted switching times. One might, on the basis of the discrepancy, even question whether the sensory and motor tasks are truly representative of the sensory-motor processes in the combined task. However, Faisal and Wolpert (2009), using the same task, used similar analyses as we report in section “Optimal switching times,” measuring the proportion of trials where combined task error was within a certain range of the minimum achievable sensory-motor uncertainty. Our results are very similar to those of Faisal and Wolpert (2009), who inferred from their data that subjects showed near optimal performance in choosing switching times.

Despite the questions that can be asked regarding the optimal control analyses, we’d like to stress the relevance of the analyses. As stated in the Introduction, shorter switching times in patients do not need to be an expression of disinhibition. They may represent an adjustment to their increased motor uncertainty and longer movement times. Hence optimal control analyses provide a means to evaluate the change as compensatory in nature or pathological. Recent data showing faster movement initiation but slower movement speed in PD patients compared to controls (Fooken et al., 2022), led the authors to ask the very same question: is it compensation or impulsivity?

### Switching time variability

In our earlier work (Sengupta et al., 2018) we found that the variability in switching time in the sensory-motor tradeoff task is modulated by the predicted minimal sensory-motor uncertainty and by the shallowness of the valley (that is the effect of variability in switching time on sensory-motor uncertainty). Both these factors are computed from the models of sensory and motor uncertainty. They can be compared with two loss functions that are used in the analysis of sensory-motor learning – one that minimizes error, relevant to error-feedback learning, and another that maximizes success, relevant to reinforcement-based learning (Cashaback et al., 2017; Kim et al., 2019). Shallowness of the valley resembles the loss function that minimizes error and predicted minimal sensory-motor uncertainty can be compared with the loss function that maximizes success.

First, we discuss the effect of predicted minimal sensory-motor uncertainty on each trial, which is an estimate of the probability of catching the ball on that trial. Our analyses revealed that, in contrast to the controls, PD patients’ variability of switching times did not show any correlation with predicted minimal sensory-motor uncertainty. This lack of correlation reveals a difference between patients and controls in exploratory behavior. Earlier studies have also shown impaired exploratory behavior in PD patients. Pekny et al. (2015) found that controls increased variability after trials with unsuccessful outcomes, whereas this increase of variability, a form of reward-dependent behavior, was attenuated in PD patients. Similarly, healthy subjects increase the variability in movement acceleration following (predetermined instead of performance-related) feedback that is biased towards punishment, while this effect is diminished following administration of a dopamine antagonist (Galea et al., 2013). We propose that the lack of correlation of our PD patients’ switching times with predicted minimal uncertainty, can be interpreted accordingly, i.e., as a probable expression of compromised reinforcement-based (sensory-motor) learning (Maia and Frank, 2011). An interesting difference between the present study and earlier work on success/reward-dependent movement variability is that we find impaired exploration in the temporal domain instead of kinematic parameters (Galea et al., 2013; Pekny et al., 2015).

Next, we consider shallowness of the valley, which is a measure of the rate of change of sensory-motor uncertainty around its minimum at the optimal switching time. From the multiple regression analyses we saw that shallowness of the valley had a significant effect on switching time variability, both in patients and controls. However, it is also clear that this is mainly driven by the ball starting from the left (Figure 7B). For balls coming from the right, PD patients show hints of a positive correlation between variability in switching time and shallowness of the valley. This behavior is in contrast with the behavior in controls and the behavior in young subjects (from our earlier work Sengupta et al., 2018). The difference with controls may be due to PD patients being more reliant on error-based learning mechanisms, as reinforcement-based learning is strongly dependent on the basal ganglia, hence is likely to be compromised (Gutierrez-Garralda et al., 2013; Todorov et al., 2019). Alternatively, variability in switching time facilitated by a shallow valley, may be a result of simplifying the control problem of finding an exact optimal solution by choosing a good enough alternative (Beck et al., 2012). On this interpretation, variability in switching time facilitated by a shallow valley does not necessarily suggest an active tendency to explore alternative solutions, as much as it suggests PD patients factored the sensory-motor error space to relax the control on switching time.

## Conclusion

Catching a moving ball is an action that Parkinson patients perform remarkably well, reducing the slowness that characterizes a similar movement to a non-moving target (Majsak et al., 1998; Bieńkiewicz et al., 2013, 2014). The observation is frequently cited in work on internally versus externally cued movement, and in the context of rehabilitation of the parkinsonian movement disorder (e.g., Morris, 2000). The present investigation was driven by the idea that the remarkable performance in ball catching might in fact be facilitated by an element of PD pathophysiology, i.e., impaired inhibition. If that were the case, our task’s artificial separation of sensing and acting stages of ball catching should have uncovered a suboptimal tradeoff between sensing and acting, detrimental to performance. This was not what we found. We observed a non-significantly shorter switching time in patients, and a significantly smaller proportion of the normalized trial duration devoted to sensing. This ambiguous result regarding switching time, creates also some ambiguity around the interpretation of the altered switching time variability that we found in patients. The reduced switching time variability in patients can be explained by reduced reward-dependent exploration in sensory-motor learning, complementing earlier work in this area (Galea et al., 2013; Pekny et al., 2015). However, it cannot be ruled out entirely that the more limited range of switching times explored by patients is partly due to an impaired ability to withhold movement so as to allow for continued sensory sampling.

Our study has several limitations. Given the long duration of experimental sessions and there being multiple sessions, we were careful to recruit patients, based on their physician’s assessment, that would be physically and mentally able to sustain the long duration. This kept the number of participants relatively small, making the study somewhat underpowered and exploratory in nature. Another limitation is the absence of a condition without the separation of sensing and acting stages. Such a condition would have provided a baseline comparison between groups as to reaction time and catching success. Finally, the study would have been stronger with the inclusion of a reference test establishing impaired inhibition. With such a test, and with results ascertaining impaired inhibition in patients, the results of our study would have had stronger bearing on the question to what extent impaired inhibition in laboratory tasks affects natural sensory-motor function.

## Data availability statement

The raw data supporting the conclusions of this article will be made available by the authors, without undue reservation.

## Ethics statement

The studies involving human participants were reviewed and approved by Ethics committee of the Faculty of Social Sciences, Radboud University Nijmegen. The patients/participants provided their written informed consent to participate in this study.

## Author contributions

SS, WM, LS, and PP contributed to the conception and design of the study. SS conducted the experiment. SS and LS performed the analysis. SS wrote the first draft of the manuscript. All authors took part in writing the final manuscript.

## Supplementary material

Supplementary Table 1 Statistical comparisons between the regression parameters reported in Table 2.
