# Supplementary material for: Exploration of sensory-motor tradeoff behavior in Parkinson’s disease
Source: Front Hum Neurosci. 2022 Oct 25;16:951313. doi: 10.3389/fnhum.2022.951313 (PMC9642091; doi:10.3389/fnhum.2022.951313)
Supplement: Supplementary Table 1 — Statistical comparisons between the regression parameters reported in Table 2. [file Table_1.docx]

**Supplementary Information**

Supplementary Table 1: Statistical comparisons between the regression parameters reported in Table 2.

| **Comparison between left and right** | | |
| --- | --- | --- |
|  | Statistics | Inference |
| **PD** |  |  |
| Observed Intercept | t(8)=-0.59, p=0.57 | The model predicts that the switching time between the left and the right should be different, but the observed switching times (as approximated by the regression parameters) are not. |
| Observed Slope | t(8)=1.75, p=0.12 |  |
| Predicted Intercept | t(8)=8.25, p<0.0001 |  |
| Predicted Slope | t(8)=-8.30,p<0.0001 |  |
| **HC** |  |  |
| Observed Slope | t(9)=0.77,p=0.46 | The model predicts that the switching time between the left and the right should be different, but the observed switching times (as approximated by the regression parameters) are not. |
| Observed Intercept | t(9)=-0.6827,p=0.50 |  |
| Predicted Intercept | t(9)=-13.53,p<0.0001 |  |
| Predicted Slope | t(9)=13.34,p<0.0001 |  |
| **Comparison between predicted and observed** | |  |
|  | Statistics | Inference |
| **PD** |  |  |
| Left Slope | t(8)=6.10,p<0.001 | The slope is a measure of the range of switching times used the range of movement amplitudes. The intercept approximates the switching time for the zero-movement amplitude. In the condition where the ball starts from the left, the predicted slope is almost always greater than the observed slope, which suggests that the range of observed switching times was smaller than what was predicted by the optimal. |
| Left Intercept | t(8)=-0.8103,p=0.44 |  |
| Right Slope | t(8)=-7.96,p<0.0001 |  |
| Right Intercept | t(8)=4.07,p=0.0036 |  |
|  |  |  |
| **HC** |  |  |
| Left Slope | t(9)=3.37,p=0.0082 |  |
| Left Intercept | t(9)=1.61,p=0.14 |  |
| Right Slope | t(9)=-12.49,p<0.0001 |  |
| Right Intercept | t(9)=10.85,p<0.0001 |  |
| **Comparison between PD and HC** | | |
|  | Statistics | Inference |
| **Left** |  |  |
| Predicted Slope | t(17)=-1.14,p=0.27 | The optimal sensorimotor tradeoff did not predict switching times to be different between the patients and controls, but some differences were observed in the slope and intercept parameters. |
| Predicted Intercept | t(17)=-1.95,p=0.07 |  |
| Observed Slope | t(17)=3.74,p=0.002 |  |
| Observed Intercept | t(17)=-3.64,p=0.002 |  |
|  |  |  |
| **Right** |  |  |
| Predicted Slope | t(17)=-1.33,p=0.20 | The optimal sensorimotor tradeoff did not predict switching times to be different between the patients and controls. No difference was observed. |
| Predicted Intercept | t(17)=0.01, p=0.99 |  |
| Observed Slope | t(17)=0.96,p=0.35 |  |
| Observed Intercept | t(17)=-1.03,p=0.31 |  |
